# Adenylyl cyclase mRNA localizes to the posterior of polarized *DICTYOSTELIUM* cells during chemotaxis

**DOI:** 10.1186/s12860-017-0139-7

**Published:** 2017-05-25

**Authors:** Satarupa Das, Joshua M. Parker, Can Guven, Weiye Wang, Paul W. Kriebel, Wolfgang Losert, Daniel R. Larson, Carole A. Parent

**Affiliations:** 1 Laboratory of Cellular and Molecular Biology, Center for Cancer Research, 37 Convent Drive, Bldg.37/Rm2066, NCI, NIH, Bethesda, MD 20892-4256 USA; 2 Institute for Physical Science and Technology, Department of Physics, University of Maryland, College Park, MD 20742 USA; 3 Laboratory of Receptor Biology and Gene Expression, Center for Cancer Research, NCI, NIH, Bethesda, MD 20892 USA

**Keywords:** mRNA, Chemotaxis, Signal relay, *Dictyostelium*

## Abstract

**Background:**

In *Dictyostelium discoideum,* vesicular transport of the adenylyl cyclase A (ACA) to the posterior of polarized cells is essential to relay exogenous 3′,5′-cyclic adenosine monophosphate (cAMP) signals during chemotaxis and for the collective migration of cells in head-to-tail arrangements called streams.

**Results:**

Using fluorescence in situ hybridization (FISH), we discovered that the ACA mRNA is asymmetrically distributed at the posterior of polarized cells. Using both standard estimators and Monte Carlo simulation methods, we found that the ACA mRNA enrichment depends on the position of the cell within a stream, with the posterior localization of ACA mRNA being strongest for cells at the end of a stream. By monitoring the recovery of ACA-YFP after cycloheximide (CHX) treatment, we observed that ACA mRNA and newly synthesized ACA-YFP first emerge as fluorescent punctae that later accumulate to the posterior of cells. We also found that the ACA mRNA localization requires 3′ ACA cis-acting elements.

**Conclusions:**

Together, our findings suggest that the asymmetric distribution of ACA mRNA allows the local translation and accumulation of ACA protein at the posterior of cells. These data represent a novel functional role for localized translation in the relay of chemotactic signal during chemotaxis.

**Electronic supplementary material:**

The online version of this article (doi:10.1186/s12860-017-0139-7) contains supplementary material, which is available to authorized users.

## Background

The ability of cells to migrate directionally when exposed to external chemical gradients, a process known as chemotaxis, is fundamental to a wide array of biological and pathological processes. A prime example of this behavior is observed in the innate immune system when neutrophils migrate to sites of inflammation and eliminate pathogens [1]. Similarly, chemotaxis is essential for the survival of the social amoebae *Dictyostelium discoideum*, where upon starvation, these cells enter a developmental program that allows their survival against harsh environmental conditions [2]. As the signaling pathways that regulate *Dictyostelium* and neutrophil chemotaxis are highly conserved, *Dictyostelium* provides a powerful model to study the biochemical and genetic basis of directed cell migration [3]. Both neutrophils and *Dictyostelium* cells exhibit amoeboid migration that uses acto-myosin driven protrusions and contractions and low cell-surface adhesions, thereby leading to fast, dynamic and plastic migration behaviors [4]. Indeed, both cell types can reach speeds of as high as 20 μm/min. Fast, spatio-temporal regulations are therefore critical during amoeboid cell chemotaxis.

In *Dictyostelium*, chemotactic migration is initiated when chemoattractants bind to G protein-couple receptors (GPCRs) leading to the dissociation of G proteins into α- and βγ-subunits and the activation of a variety of effectors that regulate cell polarity and directed motility [5]. The activation of the adenylyl cyclase ACA, which converts ATP into cAMP, is essential to relay chemotactic signals in *Dictyostelium* and requires inputs from PI3K and TORC2 [6–8]. While some of the cAMP produced remains inside the cell to activate PKA, cAMP is also secreted and acts as a chemoattractant in an autocrine and paracrine fashion by binding to GPCRs that specifically recognize cAMP (cAMP receptor 1 (cAR1)) [9]. As cells respond to cAMP gradients and migrate directionally, they align in a head-to-tail fashion and form streams - a process that increases recruitment range during chemotaxis [10]. We found that this streaming behavior not only depends on the presence of ACA, but most remarkably, on its enrichment at the posterior of polarized cells [11, 12]. Indeed, ACA is distributed in two distinct pools in polarized cells: one is restricted to the plasma membrane, the other is localized on highly dynamic intracellular vesicles that coalesce at the posterior of polarized cells. We have shown that the spatial enrichment of ACA in multivesicular bodies at the posterior of cells and the subsequent release of their internal vesicular content are essential for streaming during chemotaxis [12]. We proposed that the asymmetric distribution of vesicular ACA provides a compartment from which cAMP is locally released from the posterior of cells to spatially attract neighboring cells.

While there are many ways to target a protein, one mechanism to achieve the polarized cellular distribution of proteins involves translation of localized mRNAs [13–15]. Over the last few years, a large number of mRNAs have been shown to distribute to specific subcellular localizations, such as neuronal axons and dendrites [16], at the vicinity of specific organelles [17, 18] and at the protrusions of fibroblasts, epithelial cells and astrocytes [19–21], and to have functional consequence in a variety of processes. In fact, the regulation of gene expression by mRNA localization would be particularly well suited in situations that require high spatio-temporal resolution. Indeed, during chemotactic migration of amoeboid cells, on-site regulation of translation would bypass the requirements for signals to be targeted to the nucleus to initiate transcription, mRNA export, cytoplasmic translation and the subsequent targeting of the protein to the proper cellular site. In addition, localized mRNA translation would potentially allow for the transcript to be translated several times to locally generate many copies of the protein. Consistent with this, we previously found that the asymmetric distribution of ACA requires de novo protein synthesis and we hypothesize that localized ACA synthesis is required to maintain the active ACA pool at the posterior of polarized cells for streaming during chemotaxis [12]. We therefore set out to determine the cellular distribution of ACA mRNA in chemotaxing and streaming cells. We used FISH, developed a novel method to quantify the cellular distribution of mRNAs and used cycloheximide treatment to assess the recovery of ACA mRNA and protein during chemotaxis.

## Results

### ACA mRNA is enriched at the posterior of polarized chemotaxing cells

**Fig. 1:**
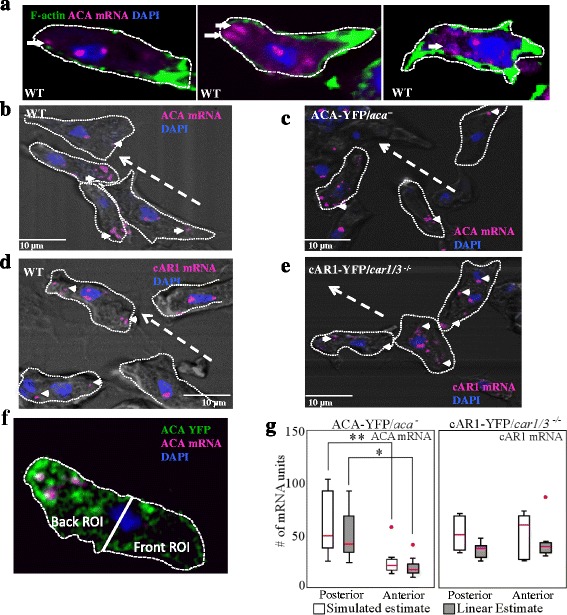
ACA mRNA is spatially localized to the posterior of polarized chemotaxing cells. **a** Representative maximum intensity projections of confocal fluorescent images of individual polarized WT cells depicting F-actin (*green*), DAPI (*blue*), and ACA mRNA (*pink*). The *white arrows* indicate the asymmetric distribution of the mRNA spots. Outline of the cells are shown in *white dashed lines*. Data are representative of at least 4 independent experiments. **b**-**c** Representative merged phase contrast and maximum intensity confocal fluorescent images depicting DAPI (*blue*) and ACA mRNA (*pink*), in WT (B) and ACA-YFP/*aca* ^*−*^ cells (C). The direction of migration of the cells (indicated by the *dashed white lines*) was determined by the position of the aggregation center towards which the cells were moving in these self-aggregating chemotaxis assays. The *white arrows* indicate the asymmetric distribution of the mRNA spots. Outline of the cells are shown in *white dashed lines*. Data are representative of at least 4 independent experiments. **d**-**e** Representative merged phase contrast and maximum intensity confocal fluorescent images depicting DAPI (*blue*) and cAR1 mRNA (*pink*) in WT (D) and cAR1-YFP/*car1/3* ^*−/−*^ (E) cells. Data are representative of at least 4 independent experiments. **f** Each cell was manually bisected, defining anterior and posterior ROI based on both the orientation towards the aggregate center and the relative posterior enrichment of ACA-YFP in the cell. **g** Simulated and linear estimates of mRNA units across cells is plotted for ACA-YFP/*aca* ^*−*^ and cAR1-YFP/*car1/3* ^*−*^ cells. The boxes show the 50% confidence region from the median (*red line*). The bars cover a region with 99% confidence level from the median. All data points beyond this confidence level are considered as outliers and are shown as red dots. The statistical significance was inferred by the t-test: * represents *p* < 0.05 and ** represents *p* < 0.01 (n_ACA_ = 33, n_cAR1_ = 27)

We examined the cellular distribution of ACA transcripts using FISH [22, 23]. We used 48 different fluorescently labeled oligonucleotide probes that span the entire *acaA* gene, thereby creating a sufficient signal-to-noise ratio to allow for mRNA detection. We acquired diffraction limited confocal image slices and reduced them to a maximum intensity projection to facilitate data analysis. As a control for these studies, we followed the distribution of cAR1 transcripts, also using 48 different fluorescently labeled oligonucleotide probes that span the entire *carA* gene. cAR1 is uniformly distributed on the plasma membrane and does not localize to intracellular vesicles [24]. We found that the FISH signals appeared in randomly distributed punctae likely representing multiple individual transcripts, within the cytoplasm of non-polarized vegetative *aca* ^*−*^ cells expressing ACA-YFP (ACA-YFP/*aca* ^*−*^) as well as *car1/3* ^*−/−*^ cells expressing cAR1-YFP (cAR1-YFP/*car1/3* ^*−/−*^ *)* (Additional file 1: Figure S1A-B). The hybridization of our ACA and cAR1 FISH probes to the *acaA* and *carA* genes was specific as no hybridization signal was observed in *aca* ^*−*^ and *car1/3* ^*−/−*^ cells, respectively (Additional file 1: Figure S1C-D). We next assessed the distribution of ACA transcripts in polarized chemotaxis competent WT and ACA-YFP/*aca* ^*−*^ cells. Whereas F-actin localized to the leading edge of WT cells, we observed that ACA mRNA was enriched at the posterior of cells (Fig. 1a). This asymmetric localization of ACA mRNA was observed in both WT and ACA-YFP/*aca* ^*−*^ cells after they were starved and pulsed for 5 h and allowed to spontaneously chemotax in a chamber slide or towards a micropipette containing cAMP (Fig. 1b and c and Additional file 2: Figure S2A&B). In contrast, both endogenous cAR1 mRNA (Fig. 1d) and cAR1-YFP mRNA (Fig. 1e) appeared uniformly distributed in the cytoplasm of polarized, chemotaxis competent cells. A similar uniform pattern was also observed using probes against actin (Additional file 1: Figure S1E). This is in agreement with previous results showing the diffused distribution of actin mRNA in *Dictyostelium* [25], as opposed to what has been reported in mammalian cells [20]. For actin, cAR1 and ACA, higher-intensity FISH spots were also observed in the nucleus, co-localizing with the DAPI signal, likely representing nascent transcripts associated with the *actin 32*, *carA*, *acaA* and genes (Fig. 1a–e & Additional file 1: Figure S1E).

We sought to quantify the cellular distribution of ACA and cAR1 mRNA transcripts in polarized chemotaxing cells. Since phase contrast images of fixed cells did not show a sharp cell boundary, we were not able to utilize active contour algorithms to define the cell boundary [26]. Furthermore, due to highly dynamic nature of cell shape changes and the presence of cell-cell contacts during streaming, we were precluded from using standard moment analysis techniques to quantify the distribution of the fluorescence intensity across the cell, as those techniques assume a well-defined and consistent geometric shape [27]. We therefore developed a “Region of Interest” (ROI) approach, where each YFP fluorescent image of a chemotaxing cell was manually segmented into anterior and posterior regions by taking the nucleus as the cell’s center (Fig. 1f), and assessed the cell’s position with respect to the aggregation center. The mRNA content of each ROI was then calculated using two methods: a linear estimate and full image simulation. Both of these methods modeled the confocal image slices as being comprised of a discrete number of fluorescent spots, which we refer to as “mRNA units”. The term “mRNA units” used in this study likely represent multiple individual mRNA transcripts as opposed to single mRNA molecules. Using peak finding algorithms on all of the images of both ACA and cAR1 mRNA and thresholding their size and intensity, we identified a characteristic smallest unit (Additional file 3: Figure S3A-B). We found that both cAR1 and ACA mRNA units were comparable in size and intensity (Additional file 3: Figure S3B). Therefore, the linear estimate of the mRNA content of an ROI is the integrated intensity inside the region divided by the intensity of a single unit. The simulated estimate, by contrast, rebuilds the image one unit at a time until the sum squared difference between the simulated image (green) and the original confocal image (red) is minimized (Additional file 3: Figure S3C-D). This rebuilding is performed multiple times to (i) obtain an average image, representing a spatial map of all of the mRNA units (Additional file 3: Figure S3E) and (ii) compute the average number of RNA units needed to reconstruct the image. Since both methods find the number of arbitrary units, not the density of units, this method of characterization depends on the accurate bisecting of the cell into anterior and posterior, but is insensitive to the accuracy of defining the cell boundary. Using both methods, we measured no preferential cellular distribution of cAR1 mRNA units in cAR1-YFP/*car1/3* ^*−/−*^ single cells (Fig. 1g). In sharp contrast, a large proportion of ACA mRNA localized to the posterior of both WT and ACA-YFP/*aca* ^*−*^ single cells (Fig. 1g).

### The asymmetric distribution of ACA mRNA and ACA protein depends on the position of the cell within a multicellular stream

**Fig. 2:**
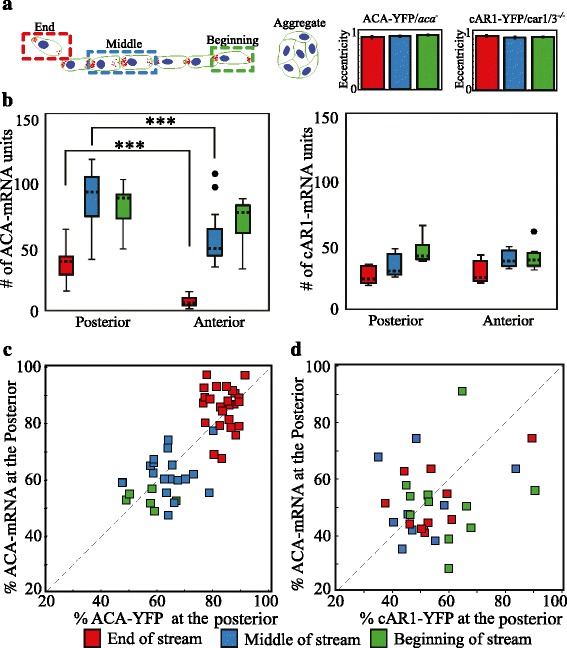
The ACA mRNA is asymmetrically distributed at the posterior of the streaming cells. **a** *Left*: Cartoon depicting the distribution of cells within a stream. Each cell was characterized as either being at the end, in the middle or at the beginning of a stream based on its position relative to the aggregate center. *Right*: Eccentricity measurement of ACA-YFP/*aca* ^*−*^ and cAR1-YFP/*car1/3* ^*−/−*^ cells at the various positions within streams (n_ACA_ = 45, n_cAR1_ = 24). **b** The simulated estimate of mRNA units across cells is plotted for ACA-YFP/*aca* ^*−*^ and cAR1-YFP/*car1/3* ^*−/−*^ cells, as a function of their position in a stream. Boxes show the 50% confidence region from the median (*dashed black line*). The bars cover a region with 99% confidence level from the median. All data points beyond this confidence level are considered as outliers and shown with *black dots*. The statistical significance is inferred by the t-test, *** represents *p* < 0.001 (*n* = 15–52). **c**-**d** The average proportion of mRNA and its corresponding protein at the posterior of cells is presented for ACA-YFP/*aca* ^*−*^ and cAR1-YFP/*car1/3* ^*−/−*^ cells (see Supplemental experimental procedure for details; *n* = 48–67)

*Dictyostelium* cells can be classified in the following groups based on their location in the line of a stream with respect to the aggregation center: at the beginning (near the aggregate), in the middle, or at the end (Fig. 2a, left panel). Using simulated images (Additional file 3: Figure S3), we quantified the number of ACA and cAR1 mRNA units localized within the cell anterior and posterior in relationship to the position of the cell in a stream. We only considered cells that were well polarized allowing manual segmentation - non-polarized cells touching the aggregation center were not taken into the analysis (see eccentricity measurements: Fig. 2a, right panel). We measured no preferential distribution of cAR1 mRNA within individual cells in all stream positions analyzed (Fig. 2b, right panel). In contrast, we measured a preferential distribution of ACA mRNA at the posterior of cells, which became stronger in cells positioned in the middle and end of streams (Fig. 2b, left panel). Indeed, we found that as cells migrate closer to an aggregation center, the ACA mRNA acquires a random cellular distribution.

In order to understand the functional significance of the ACA mRNA asymmetry, we measured the degree of ACA-YFP protein enrichment at the posterior of cells in the different stream populations. This was achieved by applying the same ROI-based image segmentation approach described earlier on the fluorescent images of ACA-YFP/*aca* ^*−*^ migrating cells and calculating the proportion of total cell pixel intensity found in the posterior (Fig. 2c and d). We observed that the extent of ACA mRNA asymmetry strongly correlates with the percentage of enriched ACA-YFP protein at the posterior of cells (Fig. 2c; Pearson’s correlation: 0.99), with cells at the end of streams showing the highest polarized distribution. This correlated polarization differed from what was seen in cAR1-YFP/*car1/3* ^*−/−*^ cells, where we measured no correlation between cAR1 mRNA and cAR1-YFP distribution (Fig. 2d; Pearson’s correlation: 0.01). As noted, we measured high eccentricity numbers for both ACA-YFP/*aca* ^*−*^ and cAR1-YFP/*car1/3* ^*−/−*^ cells (Fig. 2a, right panel), indicating that all cells within the streams were polarized to the same extent. However, only the ACA mRNA was preferentially enriched at the posterior of the cells.

### ACA protein and mRNA originate in the cytoplasm and localize to the posterior of cells as they acquire polarity

**Fig. 3:**
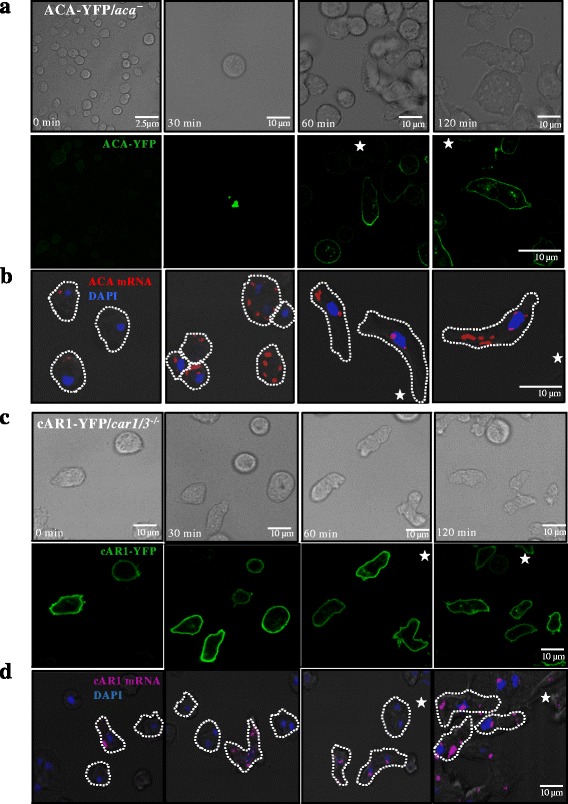
ACA translation occurs in the cytoplasm followed by localization to the posterior of the polarized cells. **a** Representative phase contrast (*upper panel*) and confocal fluorescent (*lower panel*) images of ACA-YFP/*aca* ^*−*^ cells treated with 1.6 mM CHX for 2 h. Fluorescent recovery was monitored after CHX removal. ACA-YFP protein is depicted in *green*. The *white star* indicates the location of the aggregation center. Data are representative of at least 3 independent experiments. **b** Representative maximum intensity projections of confocal fluorescent images of ACA-YFP/*aca* ^*−*^ cells depicting DAPI (*blue*) and ACA mRNA (*red*). The *white star* indicates the location of the aggregation center. Data are representative of at least 3 independent experiments. **c** Representative phase contrast (*upper panel*) and confocal fluorescent (*lower panel*) images of cAR1-YFP/*car1/3* ^*−/−*^ treated with 1.6 mM CHX for 2 h. Fluorescent recovery was monitored after CHX removal. cAR1-YFP protein is depicted in *green*. The *white star* indicates the location of the aggregation center. Data are representative of at least 3 independent experiments. **d** Representative maximum intensity projections of confocal fluorescent images depicting DAPI (*blue*) and cAR1 mRNA (*red*). The *white star* indicates the location of the aggregation center. Data are representative of at least 3 independent experiments

To visualize the appearance of newly synthesized ACA protein and mRNA in a spatiotemporal fashion, we followed the cellular distribution of the ACA protein and mRNA following recovery after translational block. We treated chemotaxis-competent ACA-YFP/*aca* ^*−*^ and cAR1-YFP/*car1/3* ^*−/−*^ cells with 1.6 mM CHX for 2 h to inhibit protein synthesis. After CHX treatment, cells were washed and ACA-YFP or cAR1-YFP protein recovery was monitored in live cells using confocal microscopy and by Western analysis. In parallel experiments, at corresponding recovery time points, samples were fixed and hybridized with FISH probes to monitor the appearance and cellular distribution of ACA and cAR1 mRNA. We found that the translational block dramatically decreased ACA-YFP levels but did not alter the expression level of cAR1-YFP (Additional file 4: Figure S4A). In addition, long term CHX treatment gave rise to round, un-polarized cells with no ACA-YFP signal (Fig. 3a; 0 min), although a few cells retained ACA mRNA signal (Fig. 3b; 0 min). As early as 30 min following CHX removal, when cells remained non-polar, newly synthesized ACA-YFP protein appeared in a vesicular pool within the cytoplasm. The recovery of ACA-YFP expression was also observed 30 min after CHX removal by Western analysis (Additional file 4: Figure S4A). One hour after recovery, ACA-YFP became enriched at the posterior of the now polarized cells and by 2 h, a strong ACA-YFP plasma membrane labeling was evident (Fig. 3a). Similarly, to the ACA protein recovery, 30 min after CHX removal the ACA mRNA signal strongly appeared in a random distribution in the cytoplasm of the non-polarized cells. As the cells acquired polarity, within 1–2 h after CHX removal, the ACA mRNA signal also became enriched at the posterior of the cells (Fig. 3b). Importantly, under these same conditions, cAR1-YFP and cAR1 mRNA signals remained unchanged throughout the entire recovery time (Fig. 3c and d). Using the quantification method described above, we estimated the number of ACA mRNA units in the anterior and posterior of cells at different times during CHX recovery. As the cells recovered and acquired polarity, we measured an increase in the number of ACA mRNA units and ACA-YFP protein at the posterior of cells within 1–2 h after CHX removal (Additional file 4: Figure S4B). Together, these findings show that following recovery from translation block, newly synthesized ACA protein and mRNA reappear and localize to the posterior of cells as they become polarized.

### ACA mRNA localization to the posterior of migrating cells requires 3′ ACA cis-acting elements

**Fig. 4:**
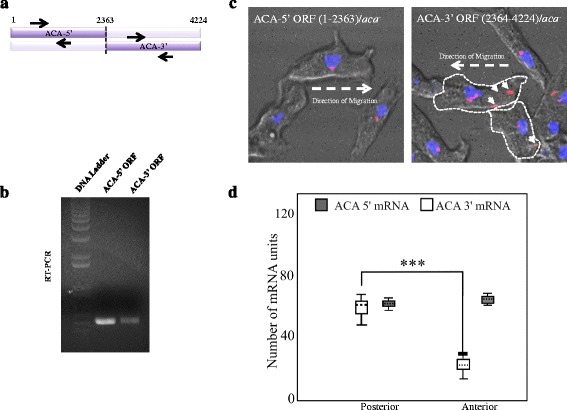
ACA mRNA localization at the posterior of migrating cells requires 3′ ACA cis-acting elements. **a** Schematic of the ACA-5’ORF or ACA-3’ORF constructs and the location of specific ACA primers used in these experiments (*black arrows*). **b** RT-PCR of the ACA-5’ORF or ACA-3’ORF cell lines using specific ACA primers. **c** Representative merged phase contrast and maximum intensity confocal fluorescent images depicting DAPI (*blue*) and ACA mRNA (*pink*) in ACA-5′ ORF/*aca* ^*−*^ and ACA-3′ ORF/*aca* ^*−*^ cells. **d** The simulated estimate of ACA mRNA units across cells is plotted for ACA-5′ ORF/*aca* ^*−*^ and ACA-3′ ORF/*aca* ^*−*^ cells. The boxes show the 50% confidence region from the median (*black dashed line*). The bars cover a region with 99% confidence level from the median. The statistical significance was inferred by the t-test: *** represents *p* < 0.01 (*n* = 10–15)

To begin to identify the cis-acting elements that regulate the distribution of the ACA mRNA, we expressed two plasmids, each harboring approximately half of the ACA mRNA open reading frame (ORF) (Fig. 4a), in *aca* ^*−*^ cells. RT-PCR analyses using specific primers confirmed that the expected transcripts were synthesized in the ACA-5′ ORF and ACA-3′ ORF cells (Fig. 4b). However, neither cell lines expressed truncated proteins (data not shown). The cellular distribution of the deleted ACA mRNAs was monitored using ACA FISH probes on chemotaxis-competent cells that were allowed to migrate on cover slips. As seen in Fig. 4c and d, while both the ACA-5′ ORF and ACA-3′ ORF cells showed a peri-nuclear distribution, the ACA-3′ ORF cells also exhibited a clear distribution at their posterior of the migrating cells. It therefore appears that elements within the 3′ end of the ACA ORF are responsible for the asymmetric distribution of the ACA mRNA during chemotaxis.

## Discussion

In *Dictyostelium*, the transmission of chemotactic signals to neighboring cells is a spatially regulated process, where the enzyme responsible for the synthesis of cAMP, ACA, is asymmetrically distributed to the posterior of streaming cells to attract neighboring cells. Furthermore, as these cells migrate, they leave behind ACA-containing vesicles, presumably secreted as exosomes, which are required for the formation of streams [12]. Constant local replenishment of ACA protein is therefore required for the streaming behavior. We now show that the ACA mRNA is also localized at the posterior of chemotaxing cells. Intriguingly, we also found that the localization of ACA mRNA at the posterior of cells is more pronounced in cells positioned in the middle and end of the streams. We envision that as cells get closer and form an aggregation center, they are exposed to saturating cAMP signals, resulting in the progressive loss of gradient sensing and the asymmetric distribution of ACA mRNA. However, cells far from the aggregation center experience a measurable cAMP gradient, allowing the maintenance of a polarized state and the ensuing enrichment of ACA mRNA at their posterior. This suggests that ACA mRNA localization requires an input signal from a functional cAMP gradient, which in turn allows the cells to polarize and migrate directionally.

In this study, we developed a novel quantification method to study ACA mRNA localization that allowed us to reliably estimate the local mRNA number density even when the mRNA punctae overlap. An ideal procedure to accomplish this would be to perform “moment analysis” on the distribution of molecules inside the cell [27], i.e. quantify the spread and anterior-posterior asymmetry by looking at the distribution of the molecules along the cell’s polarity axis. This could be done by either using the approximate locations of each point [28] or the fluorescent intensity itself. However, phase contrast images of fixed cells did not show a sharp cell boundary due to the amoeboid nature of the cells and we were therefore not able to utilize active contour algorithms to define the cell boundary [26]. Furthermore, due to the highly dynamic nature of the cell shape changes and the presence of cell-cell contacts during streaming, we were precluded from using standard moment analysis techniques to quantify the distribution of the fluorescence intensity across the cell, as those techniques assume a well-defined and consistent geometric shape [27]. We instead opted for an ROI approach, where each YFP fluorescent image of a chemotaxing cell was manually bisected into anterior and posterior regions by taking the nucleus as the cell’s center and two closed polygons were formed around the remaining portions of the cell. Which polygon represented the anterior was assessed by recording the cell’s position with respect to the aggregation center.

We explored how cells transport newly synthesized ACA mRNA and protein to their posterior. Although Actinomycin D is a commonly used transcription inhibitor, it’s GC-rich DNA binding feature compromises its ability to inhibit transcription in *Dictyostelium* cells, as *Dictyostelium*’s genome is AT rich. Instead, given the short half-life of ACA mRNA, we used CHX to visualize the localization of newly synthesized ACA mRNA as well as protein. We found that both ACA mRNA and ACA protein localize to the posterior of cells as they acquire polarity. The mechanism underlying the localized distribution of ACA mRNA at the posterior of cells remains to be determined. We did determine that cis-acting elements within the 3′ region of the ACA mRNA ORF are necessary to localize the ACA mRNA. While most mRNA localization signals localize to untranslated regions, some signals have been mapped to translated regions [29, 30]. Of the four She2p binding sites in Ash1 mRNA, only one is located in the 3′ UTR, with the remaining ones in the ORF [31]. Moreover, RNA fate can even be determined by motifs present in DNA and not RNA. For example, sequences for RNA degradation of Swi5 and Clb2 in budding yeast are encoded in *trans*, i.e. by DNA sequences in the promoter, not RNA at all [32]. In addition, during starvation in yeast, *trans* sequences coded by the promoter regulate cytoplasmic translation and RNA localization across the genome [33] and localization sequences of Oskar RNA in flies are encoded by the exon junction complex and the EJC proteins deposited on them [34]. In *Dictyostelium*, where untranslated sequences are AT rich [35, 36], we reason that ORFs, which harbor a higher GC content, may provide a better region to harbor specific mRNA localization signals. Indeed, the proper cellular localization of various mRNAs has been shown to require specific sequence and/or structural elements [37, 38]. Identification of mRNA localization elements, or “zip codes,” in a variety of systems have revealed mechanisms by which multiple sequences that form specific secondary structures can direct mRNA transport for their recognition by transport complexes [37, 38]. Although zip code-like sequences have yet to be identified in *Dictyostelium* and no examples of selective transport of mRNA and its regulated translation have been reported, we envision that similar mechanisms are at play for the precise transport and delivery of the ACA mRNA to the posterior of cells. The secondary structure of the localization sequences have also been shown to delay the passage of ribosomes along the mRNA [39], thus ensuring that the mRNA is not translated prior to its localization in a specific compartment.

## Conclusions

We show that the ACA mRNA is enriched at the posterior of polarized cells and propose that this allows the local translation and replenishment of ACA protein at the posterior of cells, where it is necessary to relay signals to neighboring cells. Our findings therefore provide the first evidence for a functional role for mRNA localization during signal relay, where the maintenance of localized protein expression is necessary to allow for fast spatio-temporal events to occur. We envision that similar mechanisms are involved in other systems. Indeed, the requirement of localized mRNA during *Dictyostelium* streaming as reported here and for fibroblast motility [20] suggests that RNA localization is an evolutionary conserved process in migrating cells.

## Methods

### Preparation of cells

WT (AX_3_), ACA-YFP/*aca* ^*−*^, and cAR1-YFP/*car1/3* ^*−*^ cells were grown in shaking cultures to ∼4 × 10^6^ cells/ml in HL5 media [11]. They were harvested by centrifugation, washed once in developmental buffer (5 mM Na_2_HPO_4_, 5 mM NaH_2_PO_4_ [pH 6.2], 2 mM MgSO_4_, 200 µmM CaCl_2_). To reach the chemotaxis-competent stage, cells were shaken at 100 rpm for 4–7 h with pulses of 75 nM cAMP every 6 min [40, 41]. The cells were then processed according to the assay performed.

### Plasmids

The ACA-5’ORF (1–2363) plasmid was generated by cloning the BclI/BbsI insert from ACA into the extrachrosomal expression plasmid pCV5, which gives high constitutive expression. The ACA-3’ORF (2364–4224) plasmid was generated using PCR and cloning into pCV5.

### Antibodies and Immunoblotting

Whole cell lysates were subjected to a 4–20% Tris-HCl SDS-PAGE analysis using the Criterion gel system and transferred to Immobilon-P (Millipore). The Immobilon-P was blotted with anti-GFP monoclonal antibody (1:5000; Babco), anti-actin (C-11; Santa Cruz Biotechnology, 1:2000) and detection was performed by chemiluminescence using a donkey anti-mouse horseradish peroxidase–coupled antibody (1:5000; GE Healthcare) or an anti-rabbit horseradish peroxidase-coupled antibody (1:10,000; GE Healthcare) and the ECL Western blotting detection reagents (GE Healthcare).

### Chemotaxis and streaming assays

The chemotaxis assays were performed as previously described [11]. Briefly, 5–7 h chemotaxis-competent cells were plated on chambered coverslips as described [11, 42] and allowed to adhere and self-stream for 30 min to 1 h. Alternatively, chemoattractant gradients were generated using a microinjector (Eppendorf) with micropipettes filled with 1 μM cAMP. The micropipette was placed in the chambered coverslips and images were captured at specified times. Once the cells started to align in streams and form aggregates, they were fixed and processed for in situ hybridization.

### Fluorescent in situ hybridization (FISH)

Vegetative or chemotaxis-competent cells were fixed in 3% paraformaldehyde (32% (wt/vol)) and permeabilized with Triton X-100 (0.1% vol/vol) in phosphate buffer. A mixture of 48 FISH DNA probes (~22 nt long) was commercially synthesized (Biosearch technologies) and processed according to the manufacturer’s protocol. Briefly, fixed cells were hybridized with the FISH probes for 4 h at 37 °C in 10% formamide in 2X saline-sodium citrate (SSC) hybridization buffer. The coverslips were washed three times with 2X and 1X SSC strengths buffers and the nuclei were stained with DAPI. The slides were imaged using a confocal microscope (Zeiss LSM 510 or 780, Carl Zeiss Inc.). Single plane images and Z stacks (1-μm confocal slice) were taken using 63 and 100X plan neofluor objectives (Carl Zeiss, Inc.) and Z stacks were arranged in maximum intensity projections.

### Simulation and quantification of the ACA mRNA localization patterns

To quantify the spatial asymmetry of the distribution of ACA mRNA, we needed a reliable estimate of the local number of mRNA units in a region of the cells. This was achieved by comparing the fluorescence intensities of the FISH signal in different cellular regions. Due to the diffraction limit, there is an inherent error in both the number estimates as well as the degree of the spatial asymmetry of ACA mRNA. We therefore obtained the mRNA number estimates using two distinct and complimentary methods. This allowed us to not only cross-check the degree of the asymmetry, but also pin down the uncertainty in this assessment. The two different methods to quantify the mRNA distribution in the cells are described in Additional file 2: Figure S2 and in Additional file 5. The anterior/posterior polarity was determined by manual segmentation, calculating the ratio of the long and short axis of the cell length in the direction of the stream towards an aggregate center. The extent of polarization of the cells was calculated using eccentricity equation epsilon = sqrt (1 - b^2 / a^2). A value of 1 indicates a parabolic or polarized cell shape and a value of 0 indicates a circle or non-polarized cell shape. During this process, we did not take into account cells whose boundaries could not be distinguished from each other in a stream.

### Cycloheximide recovery

Cells were differentiated as described for 4 h in shaking flasks containing 2 × 10^7^ cells/ml. At the end of 4 h, 1.6 mM CHX was added to the cells in shaking flask for an additional 2 h to inhibit protein synthesis. Cells were then harvested and washed to remove traces of the drug and resuspended in phosphate buffer. The cells were plated and the recovery of fluorescence was monitored at different time points by imaging using confocal microscopy. In a parallel set of experiments, cells were fixed and processed for in situ hybridization, as described. For western blot analysis, 2.7 × 10^6^ cells were harvested at various time points and resuspended in Laemmli buffer [43]. Whole cell lysates were subjected to a 4–20% Tris-HCl SDS-PAGE as described above.

## Additional files

Additional file 1: Figure S1. ACA and cAR1 mRNAs are randomly distributed in vegetative cells. A. Merge phase contrast and maximum intensity projections of confocal fluorescent images of vegetative ACA-YFP/aca- cells depicting DAPI (nucleus) and ACA mRNA (pink). B. Merge phase contrast and maximum intensity projections of confocal fluorescent images of vegetative cAR1-YFP/*car1/3−/−* cells depicting DAPI (nucleus) and cAR1 mRNA (pink). C&D. Merge phase contrast and maximum intensity projections of confocal fluorescent images of 5 h differentiated *aca-* (C) or *car1/3−/−* cells (D) depicting DAPI (nucleus) and ACA or cAR1 mRNA (pink). E. Merge phase contrast and maximum intensity projections of confocal fluorescent images of 5 h differentiated wild type AX2 cells depicting DAPI (nucleus) and actin 32 mRNA (red). The data are representative of three independent experiments. (PDF 2539 kb)

Additional file 2: Figure S2. ACA-YFP and ACA mRNA localization in chemotaxing cells. A. Representative maximum intensity projections of confocal fluorescent images of ACAYFP/*aca-* cells in natural streams, where there is significant dynamic changes in polarized states. ACA-YFP is depicted in green, ACA mRNA is in red and nucleus is in blue. The direction of migration is shown by the white arrow. The small yellow arrows highlight the posterior localization of the ACA mRNA signal. B. Representative maximum intensity projections of confocal fluorescent images of ACAYFP/*aca-* cells migrating towards a micropipette containing cAMP (yellow star). See panel A for details. (PDF 446 kb)

Additional file 3: Figure S3. Simulation and quantification of spatial ACA mRNA localization patterns. A. For each image, a peak finding routine was run on the mRNA florescent channel (left). Isolated spots were identified by thresholding their size and intensity (right). B. Peaks were fit to Gaussian point spread functions. The resulting distributions were thresholded from above until fine, unimodal distributions remained for the two fit parameters. The mean of these distributions were termed as “units”. Both ACA and cAR1mRNA showed comparable parameters. C. The sequential images from a single iteration of the image simulation procedure performed on the mRNA fluorescent channel. Areas of yellow represent agreement. D. The number of units in a particular image was determined by minimizing the squared different between the approximated image and the original. This is equivalent to minimizing the chi-square parameter of the fit. E. After performing the procedure multiple times, the average image is calculated and used for quantification. (PDF 1899 kb)

Additional file 4: Figure S4. Loss of ACA-YFP but not cAR1-YFP after CHX treatment. A. Western analysis showing protein levels of ACA-YFP from ACA-YFP/*aca −* cells or cAR1-YFP from cAR1-YFP/*car1/3−/−* cells in the presence of 1.6 mM CHX and during the recovery time points. DMSO-treated cells were used as control for this experiment. Representative data of two independent experiments are shown. B. The simulated estimate of ACA mRNA units and % ACA-YFP average fluorescence intensities 60 and 120 min after CHX removal across cells is plotted for ACA-YFP/*aca*cells. The box shows the 50% confidence region from the median (red line). The bars cover a region with 99% confidence level from the median. All data points beyond this confidence level are considered as outliers and shown with red dots. The statistical significance is inferred by the t-test, * represents *p* < 0.05 and ** represents *p* < 0.01. The data excludes the 0 and 30 min time point as these cells are not polarized, *n* = 12–22. (PDF 2659 kb)

Additional file 5: Supplementary file 1: Supplemental Experimental Procedures. (PDF 32 kb)

## Abbreviations

ACA: Adenylyl cyclase A
cAMP: 3′,5′-cyclic adenosine monophosphate
CHX: Cychoheximide
FISH: Fluorescence in situ hybridization
GPCR: G protein-coupled receptor
ORF: Open reading frame
