# Supplementary figures and images for: Adenylyl cyclase mRNA localizes to the posterior of polarized DICTYOSTELIUM cells during chemotaxis
Source: BMC Cell Biol. 2017 May 25;18:23. doi: 10.1186/s12860-017-0139-7 (PMC5445419; doi:10.1186/s12860-017-0139-7)

**A**

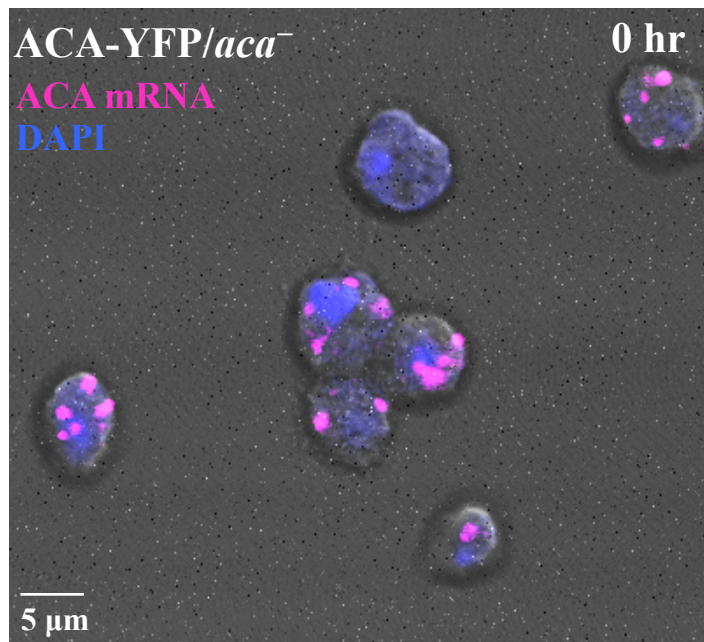

**B**

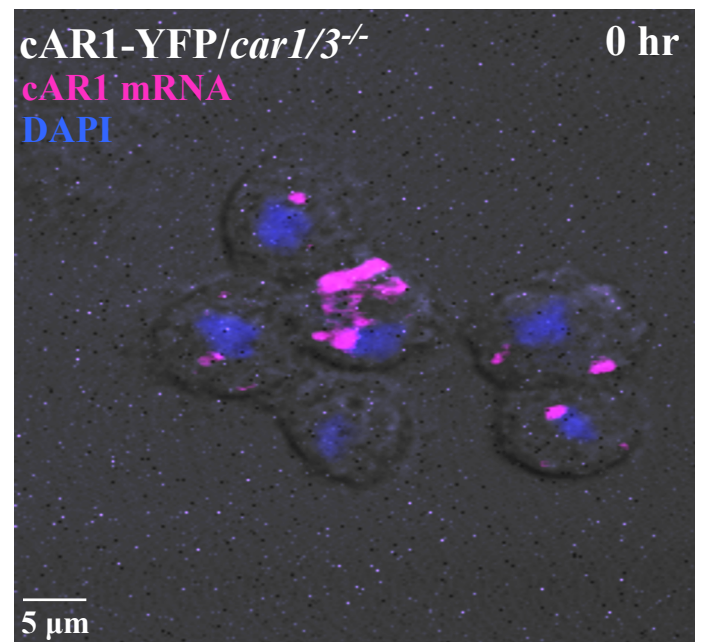

**C**

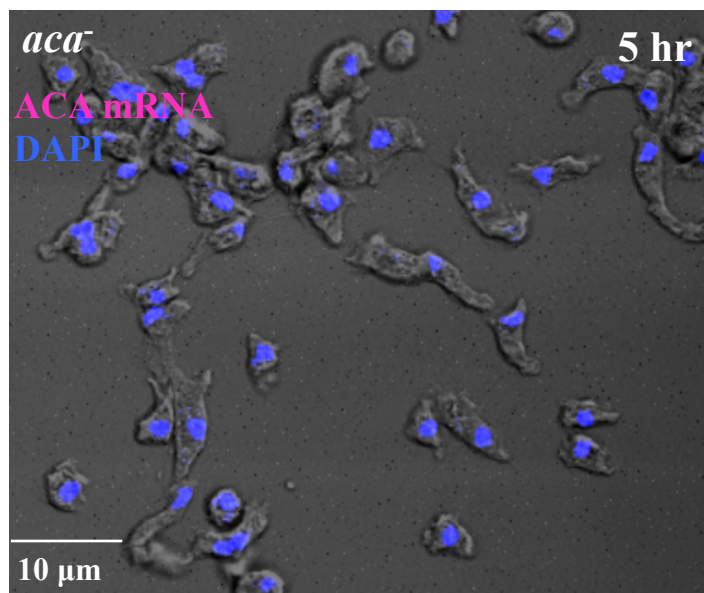

**D**

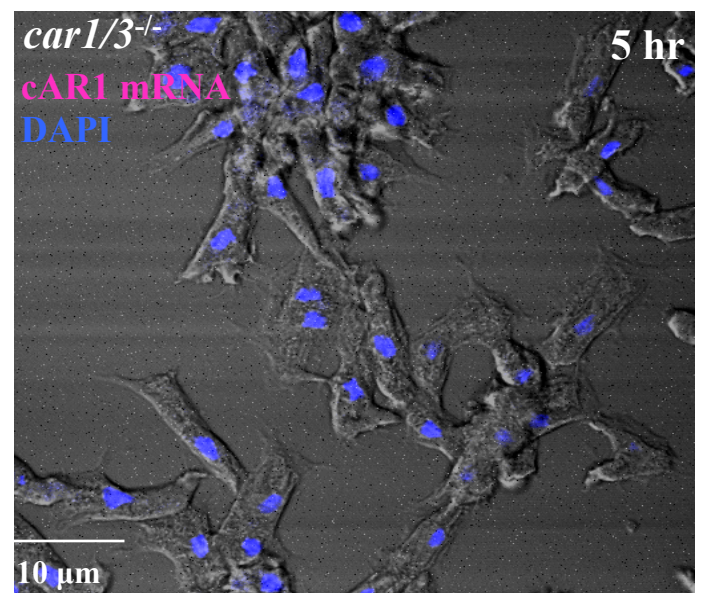

**E**

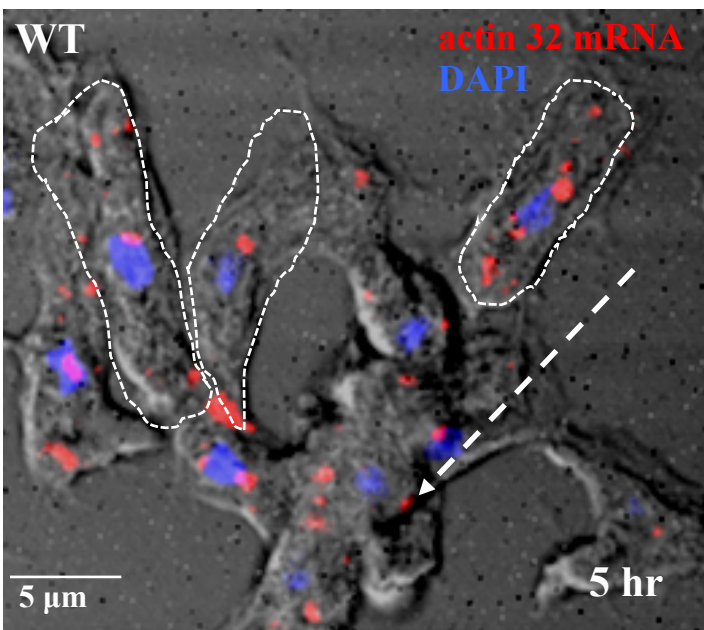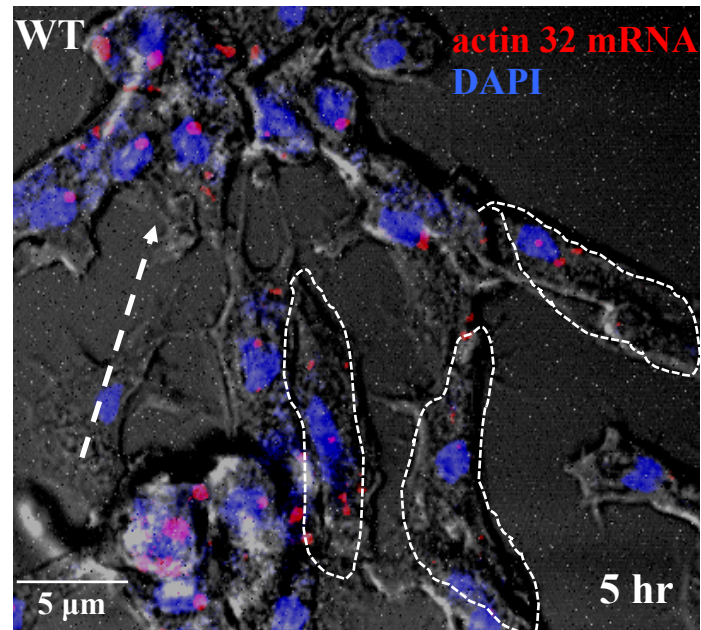

Supplement: Supplementary file 1 — ACA and cAR1 mRNAs are randomly distributed in vegetative cells. A. Merge phase contrast and maximum intensity projections of confocal fluorescent images of vegetative ACA-YFP/aca- cells depicting DAPI (nucleus) and ACA mRNA (pink). B. Merge phase contrast and maximum intensity projections of confocal fluorescent images of vegetative cAR1-YFP/car1/3−/− cells depicting DAPI (nucleus) and cAR1 mRNA (pink). C&D. Merge phase contrast and maximum intensity projections of confocal fluorescent images of 5 h differentiated aca- (C) or car1/3−/− cells (D) depicting DAPI (nucleus) and ACA or cAR1 mRNA (pink). E. Merge phase contrast and maximum intensity projections of confocal fluorescent images of 5 h differentiated wild type AX2 cells depicting DAPI (nucleus) and actin 32 mRNA (red). The data are representative of three independent experiments. (PDF 2539 kb) [file 12860_2017_139_MOESM1_ESM.pdf]

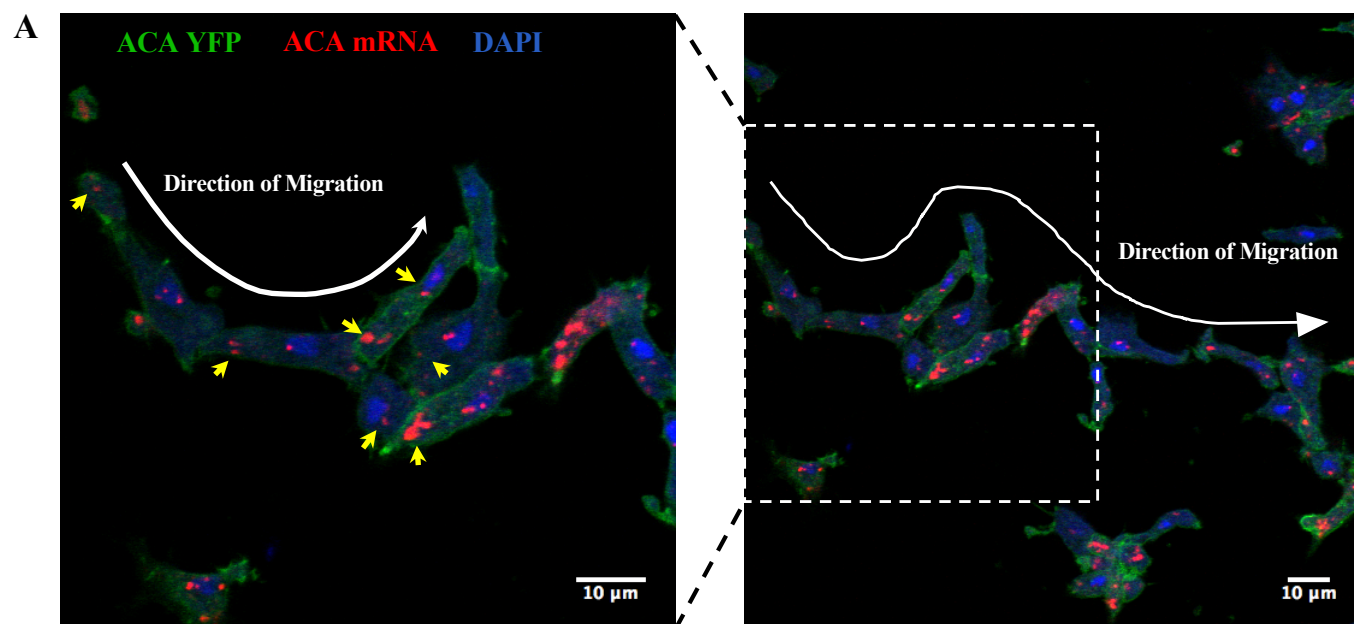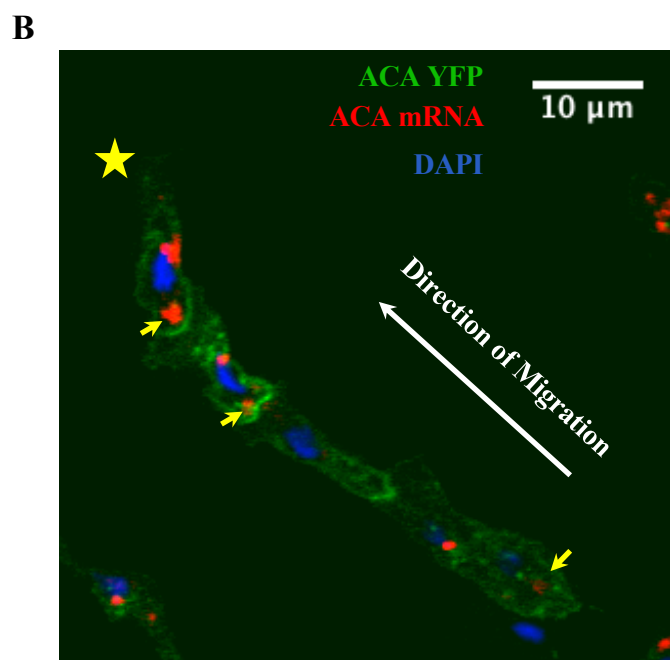

Supplement: Supplementary file 2 — ACA-YFP and ACA mRNA localization in chemotaxing cells. A. Representative maximum intensity projections of confocal fluorescent images of ACAYFP/aca- cells in natural streams, where there is significant dynamic changes in polarized states. ACA-YFP is depicted in green, ACA mRNA is in red and nucleus is in blue. The direction of migration is shown by the white arrow. The small yellow arrows highlight the posterior localization of the ACA mRNA signal. B. Representative maximum intensity projections of confocal fluorescent images of ACAYFP/aca- cells migrating towards a micropipette containing cAMP (yellow star). See panel A for details. (PDF 446 kb) [file 12860_2017_139_MOESM2_ESM.pdf]

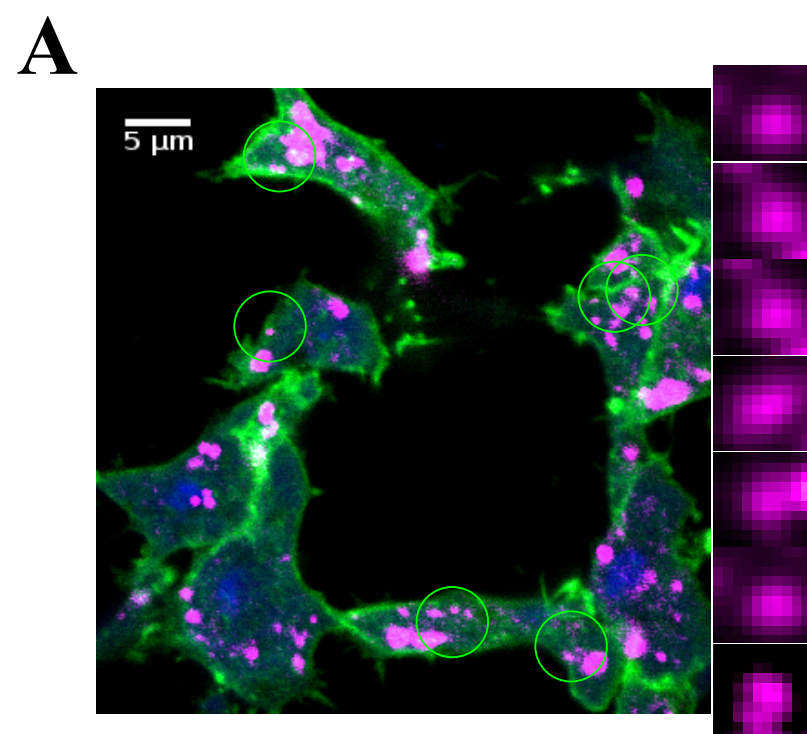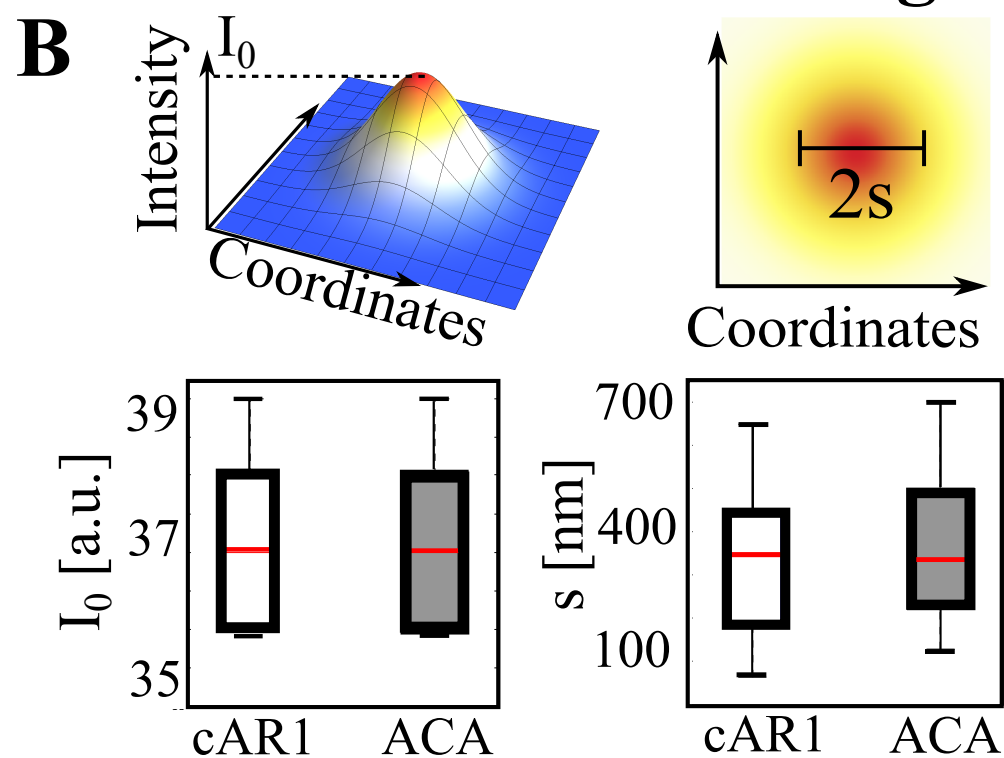

**C**      ■ Original      ■ Simulated

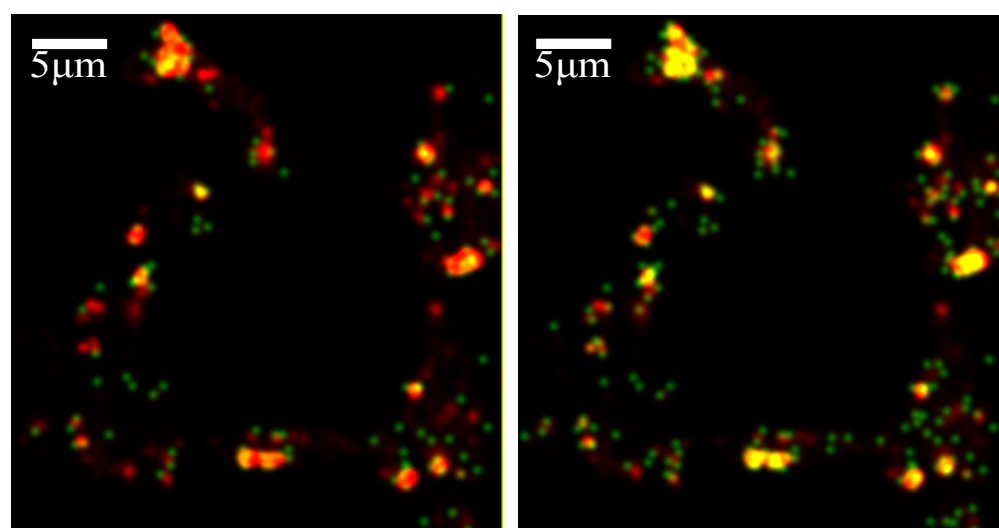

$N = 100$

$N = 250$

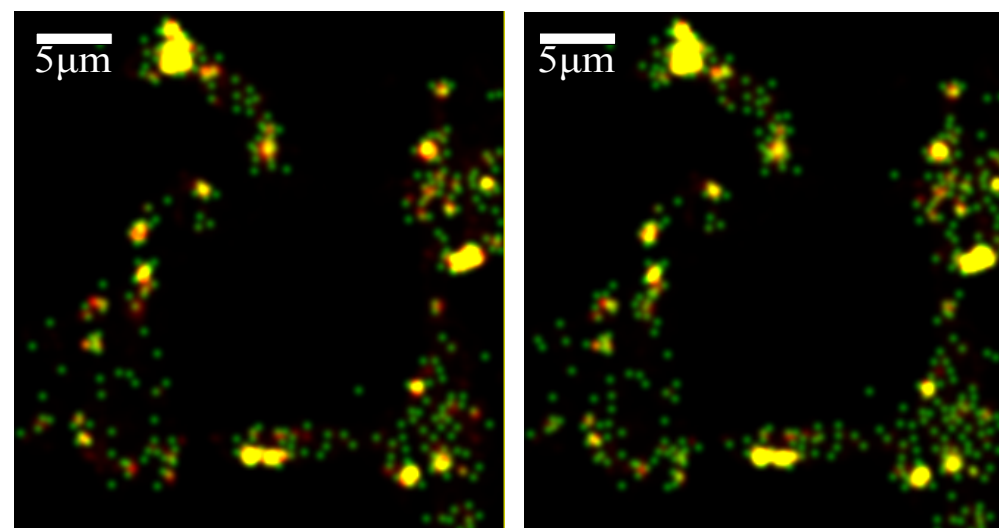

$N = 450$

$N = 550$

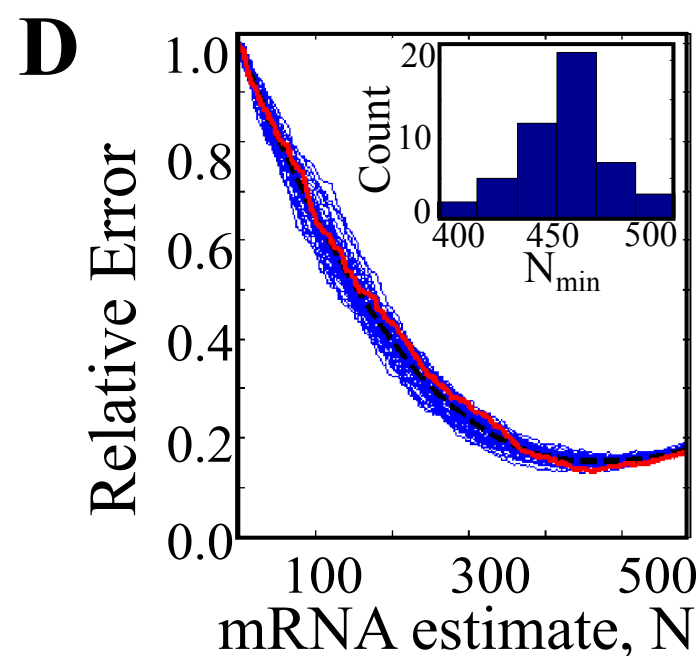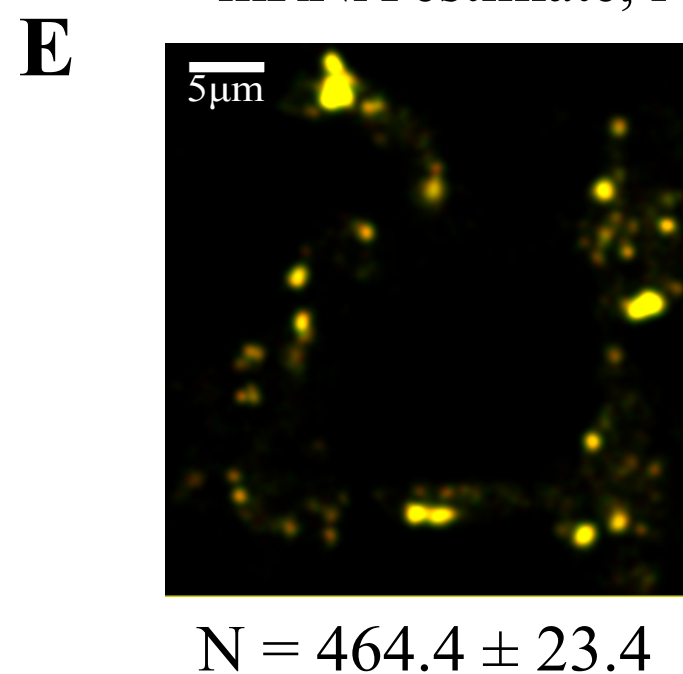

Supplement: Supplementary file 3 — Simulation and quantification of spatial ACA mRNA localization patterns. A. For each image, a peak finding routine was run on the mRNA florescent channel (left). Isolated spots were identified by thresholding their size and intensity (right). B. Peaks were fit to Gaussian point spread functions. The resulting distributions were thresholded from above until fine, unimodal distributions remained for the two fit parameters. The mean of these distributions were termed as “units”. Both ACA and cAR1mRNA showed comparable parameters. C. The sequential images from a single iteration of the image simulation procedure performed on the mRNA fluorescent channel. Areas of yellow represent agreement. D. The number of units in a particular image was determined by minimizing the squared different between the approximated image and the original. This is equivalent to minimizing the chi-square parameter of the fit. E. After performing the procedure multiple times, the average image is calculated and used for quantification. (PDF 1899 kb) [file 12860_2017_139_MOESM3_ESM.pdf]

**A**

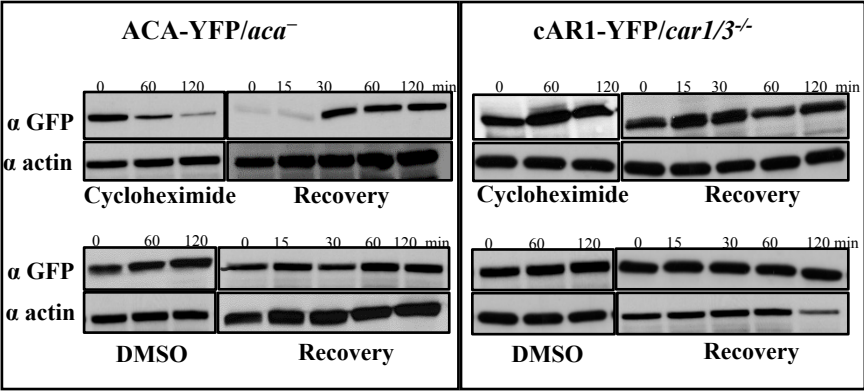

**B**

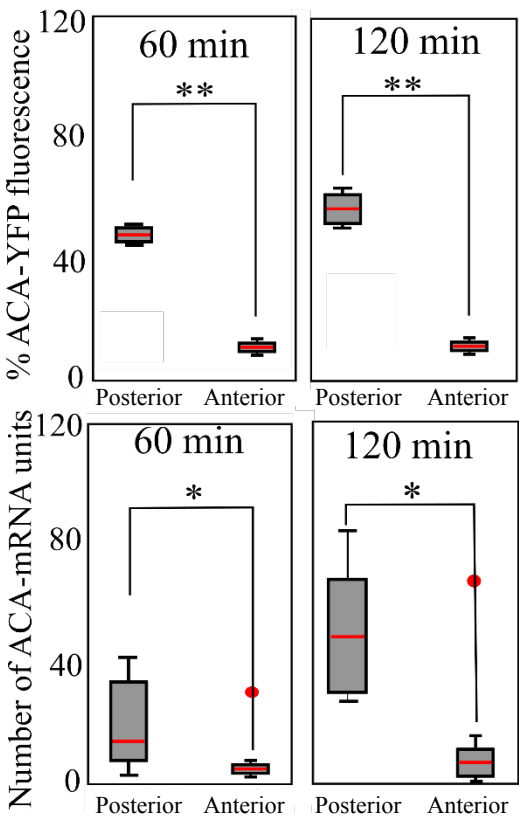

Supplement: Supplementary file 4 — Loss of ACA-YFP but not cAR1-YFP after CHX treatment. A. Western analysis showing protein levels of ACA-YFP from ACA-YFP/aca − cells or cAR1-YFP from cAR1-YFP/car1/3−/− cells in the presence of 1.6 mM CHX and during the recovery time points. DMSO-treated cells were used as control for this experiment. Representative data of two independent experiments are shown. B. The simulated estimate of ACA mRNA units and % ACA-YFP average fluorescence intensities 60 and 120 min after CHX removal across cells is plotted for ACA-YFP/acacells. The box shows the 50% confidence region from the median (red line). The bars cover a region with 99% confidence level from the median. All data points beyond this confidence level are considered as outliers and shown with red dots. The statistical significance is inferred by the t-test, * represents p < 0.05 and ** represents p < 0.01. The data excludes the 0 and 30 min time point as these cells are not polarized, n = 12–22. (PDF 2659 kb) [file 12860_2017_139_MOESM4_ESM.pdf]
