# Supplementary material for: Adenylyl cyclase mRNA localizes to the posterior of polarized DICTYOSTELIUM cells during chemotaxis
Source: BMC Cell Biol. 2017 May 25;18:23. doi: 10.1186/s12860-017-0139-7 (PMC5445419; doi:10.1186/s12860-017-0139-7)
Supplement: Supplementary file 5 — Supplementary file 1: Supplemental Experimental Procedures. (PDF 32 kb) [file 12860_2017_139_MOESM5_ESM.pdf]

SUPPLEMENTAL INFORMATION

Supplemental Experimental Procedures

Simulation and quantification of the ACA mRNA localization patterns

Two distinct and complementary methods were used to obtain the mRNA number estimates. In the first method, simple integrated intensity measurements were used to quantify the spatial distribution of mRNAs. We estimated the ACA and cAR1 mRNA intensity in a region in terms of Gaussian point spread function (i.e. units) with a peak value, I_o, and a spread value, s, representing at most a few mRNA [1, 2]. To find candidate variables for the mRNA units, we located the peak intensities in the images to find local bright regions and fit 2-dimensional Gaussian point spread functions to each one. By thresholding the distributions of their spatial size and absolute intensity, we achieved finely peaked, uni-modal distributions of I_o and s representing 274 isolated units (Fig. S2A&B). We then estimated the number of points in an ROI by dividing the integrated fluorescent intensity in that region with the total intensity of a single unit, $I_t = 2\pi s^2 I_0$. The second method involved deconvolving the image with sets of said subunits via Monte Carlo simulations. To simulate the pattern, we used the mean values for I_o and s in the following "accept/reject" Monte Carlo pattern formation procedure:

1) Calculate the maximum fluorescent intensity in the image, I_{max}.

2) Pick a random location in the image and measure the total fluorescent intensity at that location, I_{local}.

3) Draw a random number, r, which is between 0 and 1. If I_{local} is greater than $r \times I_{max}$, then consider that point to be a location of a subunit and subtract a subunit's intensity profile from that location.

4) Repeat until no suitable locations remain

This technique is a simple modification to pattern formation simulation techniques utilized in the analysis of spatial point patterns [3, 4]. Performed once, this procedure provides an estimated underlying pattern of mRNA subunits that created the image. A repeat of this procedure provides subtly different locations for mRNAs, as well as a different total number present (Fig. S2C), representing the variety of configurations that still correspond to a good match with the original image. By repeating this process multiple times, the number of subunits in an ROI is therefore estimated by the mean number of points placed in the ROI during the various simulations and the uncertainty is given by the standard error (Fig. S2D&E). This procedure therefore distinguishes itself from strict peak localization in that it does not require the mRNA to be well separated to infer the local number density. Thus, there are two possible procedures that can provide us with reliable estimates of the local number of mRNA subunits throughout the fluorescent image data set. In this paper, we only use the simulated estimate procedure. As seen in (Fig. 1G), both estimates yield comparable results.

REFERENCES

1. Trcek T, Chao JA, Larson DR, Park HY, Zenklusen D, Shenoy SM, Singer RH: **Single-mRNA counting using fluorescent in situ hybridization in budding yeast**. *Nat Protoc* 2012, **7**(2):408-419.
2. Deschout H, Cella Zanacchi F, Mlodzianoski M, Diaspro A, Bewersdorf J, Hess ST, Braeckmans K: **Precisely and accurately localizing single emitters in fluorescence microscopy**. *Nat Methods* 2014, **11**(3):253-266.
3. Wiegand T, A. Moloney K: **Rings, circles, and null-models for point pattern analysis in ecology**. *Oikos* 2004, **104**(2):209-229.
4. Illian J: **Statistical analysis and modelling of spatial point patterns**. Chichester, England ; Hoboken, NJ: John Wiley; 2008.
